# Health facilities’ readiness for safe surgical care provision in Uganda and the Eastern Democratic Republic of Congo during Ebola and COVID-19 era

**DOI:** 10.1186/s12913-021-06870-x

**Published:** 2021-08-18

**Authors:** Franck Katembo Sikakulya, Robinson Ssebuufu, Albert Ahuka Ona Longombe, Xaviour Francis Okedi, Michel Kalongo Ilumbulumbu, Moise Muhindo Valimungighe, Furaha Nzanzu Blaise Pascal, Bienfait Mumbere Vahwere, Simon Binezero Mambo, Yusuf Mulumba, Anderson Muhindo Muhasa Muyisa, Fatuma Djuma Sonia, John Sekabira, Jane O. Fualal, Patrick Kyamanywa

**Affiliations:** 1 Faculty of Clinical Medicine and Dentistry, Department of Surgery, Kampala International University Western Campus, Ishaka-Bushenyi, Uganda; 2 Faculty of Medicine, Université Catholique du Graben, Butembo, Democratic Republic of the Congo; 3 Department of General Surgery, Université de Kisangani, Kisangani, Democratic Republic of the Congo; 4 Epidemiological Surveillance, Beni, Democratic Republic of the Congo; 5 Department of General Surgery, University Abomey-Calavi, Abomey-Calavi, Benin; 6 Department of Anesthesia and Intensive care, College of Medicine, University of Malawi, Blantyre, Malawi; 7 Youth Alliance for Reproductive Health, Goma, Democratic Republic of the Congo; 8 Biostatistics, Cancer Institute, Makerere University, Kampala, Uganda; 9 Department of Surgery, Mulago Hospital, Kampala, Uganda

**Keywords:** Health facilities, Safe surgical care provision, Readiness, Ebola, COVID-19

## Abstract

**Objective:**

This study aimed to assess health facilities’ readiness to provide safe surgical care during Ebola and COVID-19 era in Uganda and in the Eastern DR Congo.

**Methods:**

A cross-sectional study was conducted in selected national, regional referral and general hospital facilities in Uganda and in the eastern part of DR Congo from 1st August 2020 to 30th October 2020. Data was analysed using Stata version 15.

**Results:**

The participation rate was of 37.5 % (72/192) for both countries. None of the hospitals fulfilled the readiness criteria for safe surgical care provision in both countries. The mean bed capacity of participating health facilities (HF) was 184 in Eastern DR Congo and 274 in Uganda with an average surgical ward bed capacity of 22.3 % (41/184) and 20.4 % (56/274) respectively. The mean number of operating rooms was 2 and 3 in Eastern DR Congo and Uganda respectively. Nine hospitals (12.5 %) reported being able to test for Ebola and 25 (34.7 %) being able to test for COVID-19. Postponing of elective surgeries was reported by 10 (13.9) participating hospitals. Only 7 (9.7 %) hospitals reported having a specific operating room for suspect or confirmed cases of Ebola or COVID-19. Appropriate Personal Protection Equipment (PPE) was reported to be available in 60 (83.3 %) hospitals. Most of the staff had appropriate training on donning and doffing of PPE 40 (55.6 %). Specific teams and protocols for safe surgical care provision were reported to be present in 61 (84.7 %) and 56 (77.8 %) respectively in Uganda and Eastern DR Congo participating hospitals.

**Conclusions:**

The lack of readiness to provide safe surgical care during Ebola and COVID-19 era across the participating hospitals in both countries indicate a need for strategies to enhance health facility supplies and readiness for safe surgical provision in resource-limited settings.

## Introduction

As the COVID-19 pandemic continues to evolve and spread worldwide, health facilities are intensifying measures for protecting patients and health workers from this highly infectious disease [1]. With a varied incubation period of 2 to 14 days [2], infected asymptomatic patients, can transmit the disease to a non-infected person and this accounts for the significant ongoing community transmission [2]. The spread of the COVID-19 virus in health facilities is largely from asymptomatic patients and healthcare providers and those with mild or nonspecific respiratory syndromes, leading to a cluster of nosocomial infections [3].

With over 10 million cases confirmed worldwide (over 4 million confirmed in Africa) as of 13th July 2021, COVID-19 compounded an already complicated situation in the Eastern Democratic Republic of Congo (DRC), with both armed conflict and the highly contagious Ebola virus disease (EVD) outbreaks (from August 1, 2018 to of June 21, 2020 in the Kivu). A total of 3,317 confirmed EVD cases and a death toll of 2,287 patients including healthcare workers (HCWs) and two fatalities registered in the neighboring Uganda was reported [4, 5]. The 11th EVD outbreak was declared in the Equator Province in the western DRC last year registering 130 cases including 55 deaths [6]. The 12th EVD outbreak declared in Butembo on 7th February 2021 by the DRC Ministry of Health with a total of 12 cases, 6 deaths (case fatality rate 50 %) by its end on 3rd May 2021[6].

Several facility-based measures have been put in place to mitigate the spread of COVID-19 and its impact on the health systems. The measures include the use of personal protective equipment (PPE) when handling patients, testing, isolation and treatment of symptomatic patients, and contact tracing, in addition to quarantine of the suspected cases [1, 7]. However, since not all patients in need of surgery are being routinely tested for COVID-19 and asymptomatic patients could spread both diseases to the non-infected staff in the surgical operating rooms, it was suggested that all surgical patients should be considered as possibly positive in order to limit the contamination of healthcare workers [8]. Following the declaration of the COVID-19 pandemic in March 2020, elective surgeries were cancelled in most countries and several additional measures such as use of PPE, psychological support to all surgical teams, COVID-19 test for all patients who need an emergency surgery and others have been proposed to limit the risk of contamination among surgical patients and staff [9, 10].

It is worth noting that during the West African Ebola outbreak in 2014, one of the measures put in place was that, for surgery to be done, the caregiving team was requested to undertake a documented utility risk analysis, which included not only the perspective of the patient, but also the surrounding surgical team [11]. A similar practice would be beneficial even in the current situation especially where there is a double threat from Ebola and COVID-19. Shortages of PPEs and operating rooms have changed the way surgical diseases are managed during the COVID-19 [12], with the American College of Surgeons proposing a triage algorithm with the purpose of preserving staff, PPE, and patient care supplies; ensuring staff and patient safety; and expanding available hospital capacity during the COVID-19 pandemic [8, 13, 14].

In Africa, the College of Surgeons of East, Central and Southern Africa (COSECSA) proposed general guidelines for surgical readiness during the COVID-19 period that includes factors such as isolation of confirmed COVID-19 patients, use and application of PPE, hand hygiene, limitation of movement through the hospital and wearing of surgical masks for all confirmed cases when being transported through hospital space or in rooms [14]. Similar measures have been applied by surgical teams in different countries with varying success [15].

Worldwide, surgical care delivery to the general population has been affected by the staggering increase in the demand for medical supplies, reduced in-person medical visits, and shortages of medical protective gear [16] which has led to the delay in surgical care and follow-up of surgical patients in China, Germany and in Dubai [17]. In Africa, preparedness is challenged by the general weakness of health systems and structures such as shortage of human resources, lack of equipment and facilities and vulnerable supply chains [18]. While most governments across Africa already rely heavily on assistance from donors in the health area, the fragmented and insufficient responses have led to the creation of national public health institutes that have obliged these countries to look for ways to collaborate and work together to fight this weakness of health structures [18].

The availability of human resources, drugs, equipment and basic infrastructural amenities is crucial for providing quality health care services that meet minimum standards [19]. In high- and middle-income countries, suitable facilities and equipment, human resources, and infrastructure are available in acceptable ratios even in the district hospitals [20]. The situation is quite different in low-income countries, especially in Africa. To be able to respond appropriately to the situation and to avoid the negative impact on provision of surgical care, there is need to know the reality on the ground in terms of facilities, the necessary equipment and supplies, and the strengths and weaknesses in terms of availability of suitably qualified human resource for health for surgical care. Therefore, this study aimed to assess health facilities’ readiness to provide safe surgical care during Ebola and COVID-19 era in Uganda and in the Eastern *DR* Congo to inform about the need of health facilities for safe surgery during these outbreaks.

## Methods

### Study design and setting

 This was a cross-sectional study conducted in selected national, regional referral hospitals (R.R.H) and general hospitals (G.H.) of Uganda and in the tertiary hospitals (T.H) and general hospitals (G.H) of the Eastern DRC where surgeries are being done during Ebola and COVID-19 era. These two countries were chosen as they share a long border and have recently been simultaneously affected by Ebola and the ongoing COVID-19 pandemic; furthermore, with the past experience of viral disease outbreaks such as Marburg and Ebola, both countries are assumed to be prepared to provide safe surgical care. A total of 192 hospitals were selected out of which 158 were in Uganda and 34 hospitals in Eastern DRC. Of the 158 hospitals in Uganda, 5 are national hospitals, 14 R.R.H and 139 GH and of the 34 hospitals in the Eastern DR Congo there were 2 TH and 32 G.H.

### Study participants and recruitment

This study involved medical doctors and/or surgeons working in the selected health facilities and consented to participate in the study and responded to the questionnaire. At each participating hospital, a senior doctor (medical director or head of surgical department) was contacted through a phone call, a clear explanation of the study given and they were invited to participate in the study on behalf of their hospital.

### Data collection and instrument

The study was conducted for a period of three months from 1st August 2020 to 30 October 2020 and the participants were invited to respond to a structured questionnaire sent via WhatsApp or mail. The structured questionnaire was composed of 22 questions developed based on the elements from the COSESCA general guidelines for surgical readiness during the COVID-19 [14] and focused on several key concepts. Section A had 6 questions related to characteristics of the health facility (country, name of hospital, hospital sector, hospital bed capacity, surgical bed capacity and number of active operating rooms). Section B had 16 questions developed based on the COSESCA general guidelines for surgical readiness [14] during the COVID-19.

In section B, each of the 16 guideline- related questions contained 2 options “Yes” for ready and “No” for not ready. A health facility was said to be ready if it had fulfilled all the 16 items of the study. Otherwise, it was said “not ready”.

The questionnaire was pre-validated and piloted in two hospitals in Uganda and in Eastern DRC, whose responses were not included in this study.

All national, regional referral and general hospital facilities of Uganda and in the tertiary and general hospitals of the Eastern part of DRC were eligible to participate in the study. A judgement sampling technique was used to identify the participating hospitals. Participants selected in these hospitals were encouraged to fill the form and send it back within 1 month from the date of reception. Each hospital was required to submit one copy of the filled questionnaire which was considered for data analysis. The questionnaire used in the DRC was in French and the filled form was translated to English by one of the authors (FKS) and cross-checked for accuracy by two authors (FNBP and PK) who are fluent English and French speakers, before consideration for analysis. Data collection in each country was coordinated by one trained researcher who was in charge of identifying participant hospitals, distributing and follow-up of the structured questionnaire via e-mail and/or WhatsApp to the heads of surgical departments of the selected hospitals in the study setting.

 On receiving the questionnaire and after reading the preamble and consenting to participate, participants were directed to fill in the questionnaire which was then sent back to the country coordinator for transmission to the principal investigator for data extraction, processing and analysis.

### Data processing and analysis

The raw data was cleaned and entered into Microsoft Excel and exported into STATA version 15 used for statistical analysis (StataCorp, College Station, Texas, USA). A univariate analysis of all categorical data (characteristics of the health facility) was done and presented in figure with their frequencies, percentages and the mean and standard deviation for continuous variables. The sixteen questions on readiness were compared by country (Uganda and DRC) using the Chi-square statistics and presented in contingency table with their frequencies and percentages. Readiness to provide safe surgical care was assessed based on the fully presence of 16 elements adapted from the COSESCA general guidelines. Each hospital was scored out of 16 according to the number of items and a mean number of present items was calculated for each region. The difference between these means were assed with t-test with significance level of 5 %. The distribution of presence of the items by country and sectors was presented using quartiles.

### Ethical consideration

 Ethical clearance for the survey was obtained from the Institutional Research Ethical Committee of Kampala International University (KIU-REC-023/2020) in Uganda and the Comite d’Ethique du Nord Kivu (004/TEN/CENK/2020) in the Democratic Republic of Congo. Permission to access health facilities was obtained from all relevant local health authorities. The participation in this survey was voluntary. Participants were allowed to withdraw from the study at any time by not submitting their form online or sending an email to the principal investigator (PI) and there was no penalty for withdrawing from the study. The participants’ identities remained concealed as the form did not require any identification. Names of the participants at the selected hospitals were not required. Informed consent was obtained from all the participants.

## Results

**Fig. 1:**
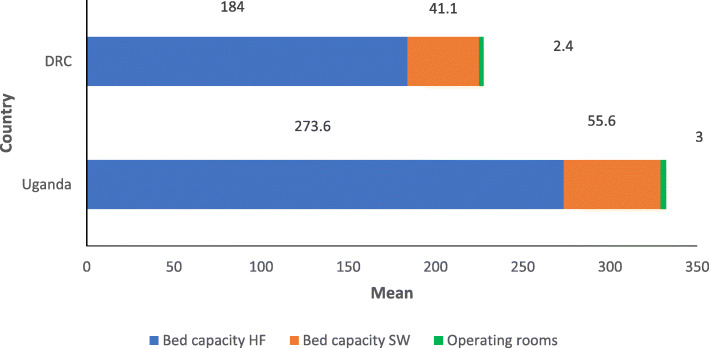
Average of hospital bed capacity, surgical ward bed capacity and operating rooms per responding hospital. HF: Heath facility. SW: Surgical ward

Out of 192 preselected hospitals in Uganda (158) and Eastern DRC (34), 72 hospitals responded to the questionnaire during data collection period with a response rate of 37.5 % for both countries. From the 72 hospitals, 47 (65.3 %) were from Uganda and 25 (34.7 %) from Eastern DRC. From the participant hospitals, 45 (62.5 %) represented public and 27 (37.5 %) private sectors respectively. The mean bed capacity of the health facilities (HF) was 184 (min: 60 and max: 500) in Eastern DRC and 274 (min: 80 and max: 1000) in Uganda. The average bed capacity on surgical ward (SW) represented 22.3 % (41/184) of the beds in the DRC and 20.4 % (56/274) in Uganda. The mean operating rooms was 2 (SD of 1.7, minimum of 1 and Maximum of 9) and 3 (SD of 1.6, minimum of 1 and Maximum of 10) in Eastern DRC and Uganda respectively (Fig. 1).

### Health facilities readiness for safe surgical care provision in Ebola and COVID-19 era

Out of 72 respondent hospitals, 9 (12.5 %) reported being able to test for Ebola and 25 (34.7 %) being able to test for COVID-19. Only 7 (9.7 %) hospitals reported to have an operating room specific for suspected or confirmed cases of Ebola or COVID-19. Team response for Ebola and COVID-19 were reported to be present for 61 (84.7 %) hospitals and provision of appropriate PPEs to personnel were reported to be available in 60 (83.3 %) hospitals. Overall, the rate of correct answer on readiness reported by the hospitals ranged from 9.7 to 84.7 % by item (Table 1).

**Table 1:** Items related to safe surgical care provision during Ebola and COVID-19 era

Variable group	Items related to readiness	The Eastern DRC ***n*** = 25(%)		Uganda ***n*** = 47(%)		Total ***n*** = 72 (%)
		Yes	No	Yes	No	Yes
Readiness	Hospital able to test for Ebola	5 (20.0)	20 (80%)	4 (8.5)	43 (91.5)	9 (12.5)
	Hospital able to test for COVID-19	5 (20.0)	20(80%)	20 (42.6)	27 (57.4)	25 (34.7)
	Postponing elective surgeries during outbreaks period	5 (20.0)	20 (80)	5 (10.6)	42 (89.4)	10 (13.9)
	Having one operating room specific for suspect or confirmed cases	1 (4.0)	24 (96)	6 (12.8)	41 (87.2)	7 (9.7)
	Training of staff on appropriate donning and doffing of PPE	9 (36.0)	16 (64)	31 (66.0)	16 (34)	40 (55.6)
	Having teams specifically for outbreaks response	21 (84.0)	4 (16)	40 (85.1)	7 (14.9)	61 (84.7)
	Having protocols specifically for outbreaks response	20 (80.0)	5 (20)	36 (76.6)	11(23.4)	56 (77.8)
	Use of checklist for suspected/confirmed patients during surgery	17 (68.0)	8 (32)	29 (61.7)	18(38.3)	46 (63.9)
	Avoid involving students/Residents in patient care of infected patients	5 (20.0)	20(80)	9 (19.1)	38 (80.9)	14 (19.4)
	Reduction of the staff number required in the hospital to preserve human resource	7 (28.0)	18 (72)	17 (36.2)	30(63.8)	24 (33.3)
	Providing appropriate PPEs to personnel	25 (100.0)	0 (00)	35 (74.5)	12(24.5)	60 (83.3)
	Having containers (disposable bag) for any used PPE	12 (48.0)	13 (52)	42 (89.4)	5(10.6)	54 (75.0)
	Disinfection of all hard surface areas regularly with 0.5% chlorine or 70% alcohol	16 (64.0)	9 (36)	14 (29.8)	33 (70.2)	30 (41.7)
	Provide psychological support to staff during this time of crisis	2 (8.0)	23(92)	27 (57.4)	20 (42.6)	29 (40.3)
	Similarity or increase on HCW’s remuneration	17 (68.0)	8 (32)	35 (74.5)	12 (24.5)	52 (72.2)
	Timely remuneration of HCW’s	14 (56.0)	11 (44)	32 (68.1)	15 (31.9)	46 (63.9)

*HCW’s* Healthcare workers

### Readiness for safe surgical care provision by country

None of the hospitals had the 16 items present and was classified as ready for safe surgical care provision in Eastern DRC and in Uganda. The mean of present items was of 8 items (50 %) (minimum of 3 and maximum of 13) in both countries, 7 items (minimum of 3 and maximum of 11) in the Eastern DRC and 8 (minimum of 4 and maximum of 13) in Ugandan. The mean number of items in the public sector was of 8 (minimum of 3 and maximum of 13) and in the private sector of 8 (minimum of 4 and 13 as maximum). The difference between countries or sectors was not significant (*p*-value > 0.05). The table number 2 below give the repartition of HF according to number of items (Table 2).

**Table 2:** Overall Health facilities' readiness for safe surgical care provision by country (*n* = 72)

Hospital Readiness	Eastern DRC	Uganda
Not ready (15 or less)	25	47
Ready (16 items)	0	0
Total	**25**	**47**

### Quartile distribution of items by country and sector of hospitals

From the 16 items, two 2 hospitals (4.3 %) in Uganda (1 private and 1 public) had more than 75 % of the items needed. Most of hospitals had at least 50 % of items for readiness. There was no difference between countries or sectors (p:>0.05) (Table 3).

**Table 3:** Quartile distribution of the readiness by country and sector of hospitals

Number of items	25 %	50 %	75 %	> 75 %	Total	*P*-value
Country, n (%)						
DRC	3 (12.0)	14 (56.0)	8 (32.0)	0 (0.0)	**25 (100.0)**	
Uganda	1 (2.1)	21 (44.7)	23 (48.9)	2 (4.3)	**47 (100.0)**	0.142
Total (%)	4 (5.6)	35 (48.6)	31 (43.1)	2 (2.8)	**72 (100.0)**	
Sector of the hospital, n (%)						
Private	1 (3.7)	13 (48.2)	12 (44.4)	1 (3.7)	**27 (100)**	
Public	3 (6.7)	22 (48.9)	19 (42.2)	1 (2.2)	**45 (100)**	0.936
Total (%)	4 (5.6)	35 (48.6)	31 (43.1)	2 (2.8)	**72 (100.0)**	

## Discussion

This study assessed the readiness of the health facilities of Eastern DRC and Uganda to provide safe surgery during the Ebola and COVID-19 outbreaks. We derivate an assessment tool from the COSESCA guidelines for safe surgical care preparedness during COVID-19 and added items related to Ebola outbreak since the two outbreaks occurred simultaneously in both countries [14].

For a health facility to be considered ready, it has to: (a) develop a clear plan for providing essential operations during the pandemic; (b) develop strategies to decrease exposure of health care staff and (c) develop capacity to conserve PPE and consumables [14]. General weakness of health system is challenging the preparedness of health facilities to face outbreaks in Africa [18].

This study reported a lack readiness to provide safe surgical care during Ebola and COVID-19 era. None of the hospitals registered was theoretically ready to provide safe surgical care in Eastern DRC and Uganda in Ebola and COVID-19. This highlights a high-risk practice with possibly increased morbidity and mortality during these outbreaks. This result is similar to findings in a study done by Spiegel et al. [21] on surgical availability and readiness in 8 African countries in which Uganda was reported to have a higher readiness score for basic surgery compared to other countries. However, Kibuule et al. reported a lack of preparedness of Uganda’s health facilities to fight EVD despite having faced previous outbreaks or EVD and Murburg and neighboring DRC which was facing EVD at the time of data collection [22].

The average bed capacity varied across the two countries in this study. Hospitals in Uganda reported having a lower average of bed capacity on surgical ward than those in the Eastern DRC. However studies assessing public hospital surgical capacity in the DRC [23] and Uganda [24] have shown the average number of hospital beds to be 150 (2.5 operating rooms) and 257.1(2.63 operating rooms) respectively. The study found that 90.3 % of hospitals reported not having a separate theatre room specific for suspect or confirmed cases of Ebola and/or COVID-19. This increases the potential risk for infectious disease dissemination among patients and HCWs, since it was compounded by the lack of testing for Ebola and COVID-19 in most respondent hospitals. It is a good practice for each hospital providing surgery to have a separate operating room specific for confirmed or suspected COVID-19 or Ebola cases [14, 25].

According to the International Monetary Fund (IMF), LIC’s reported financial shortage in most of the hospitals [26] and this survey has found that hospitals in Uganda and Eastern DRC hospitals are facing financial shortages impeding provision of safe surgery. However, teams, protocols and appropriate PPE’s to HCWs were reported to be available in most hospitals of Uganda and Eastern DRC. This can be due to the fact that both countries have participated in infectious disease control related campaigns in response to repeated outbreaks of Ebola and Marburg Outbreaks [27]. The level of training of staff on appropriate donning and doffing of PPE was found to be inadequate in both countries despite the recommendation that guidance and training should be provided immediately to HCWs so as to make the best use of their technical and clinical skills [14, 28]. Despite having appropriate PPEs in both countries and specific teams to manage the suspected or confirmed cases of Ebola and or COVID-19, there was use of unqualified health workers such as students reported in both countries. It has been recommended that during outbreaks, only qualified health workers or specifically prepared teams be used to minimize the risk of nosocomial infection [14, 29]. In the present study there was no reduction of surgical team personnel although the American society of surgeons and COSESCA recommended reduction of intraoperative personnel and use of checklists to prevent intraoperative infection-transmission [13, 14].

This study found a lack of provision of psychological support to healthcare workers. However, it has been reported that HCWs on duty during periods of outbreaks need psychological support as they have been reported to have significant anxieties while providing care outside of their normal scope of practice or working beyond their area of competence [30]. This anxiety must be acknowledged and managed.

### Study limitations

Participation of all pre-selected hospitals in Uganda and Eastern DRC was not possible despite efforts made to get responses from the participants to whom we sent the questionnaire and several reminders to them to send back the filled questionnaire, thus the findings from this survey will not be generalized to all the hospitals in the two countries. This study is not to illustrate the effectiveness or ineffectiveness of management of hospitals. Furthermore, this study was conducted during the lockdown of the first wave of COVID-19 when the elective surgeries had been suspended and there was no COVID vaccine available in Uganda and DR Congo. Due to the COVID19-related restriction on travel and interaction it was not possible to conduct face-to-face qualitative interviews to gain deeper understanding of the operations of the hospitals. Therefore, a word format questionnaire sent online was used to allow the researchers to explore the surgical readiness of hospitals in Uganda and Eastern DRC.

## Conclusions

The findings suggest a lack of readiness for safe surgical care provision among hospitals in both countries in terms of equipment, supplies to limit the exposure of HCWs, remuneration and support of the HCWs during the Ebola and COVID-19 era. There is urgent need for interventions by the governments and non-governmental organisations to work together in improving health worker safety, facility supplies and funding to enhance the readiness for safe surgical provision in the two countries. The readiness process must be constantly monitored and the surgical societies should champion the advocacy for adequate supplies and better remuneration of HCWs.

## Data Availability

The data used to obtain the findings is available from the corresponding author FKS and the author YM on a reasonable request.

## Abbreviations

CENK: Comite d’Ethique du Nord Kivu
COSECSA: College of Surgeons of East, Central and Southern Africa
DRC: Democratic Republic of Congo
EVD: Ebola virus disease
HCW: Health Care Workers
IMF: International Monetary Fund
IREC: Institutional Research Ethical Committee
KIU: Kampala International University
LIC: Low Income Country
PPE: Personal protective equipment
